# The Effect of Salacia Reticulata Extract Biscuits on Blood Sugar Control of Type 2 Diabetes Mellitus Patients: A Two-Period, Two-Sequence, Crossover, Randomized, Triple-Blind, Placebo-Controlled, Clinical Trial

**DOI:** 10.7759/cureus.45921

**Published:** 2023-09-25

**Authors:** Sisira Siribaddana, Arjuna Medagama, Nadeesha Wickramasinghe, Nipuna M Siribaddana, Suneth Agampodi, Devaka Fernando

**Affiliations:** 1 Medicine, Faculty of Medicine and Allied Sciences, Rajarata University of Sri Lanka, Anuradhapura, LKA; 2 Medicine, Teaching Hospital Anuradhapura, Anuradhapura, LKA; 3 Medicine, University of Peradeniya, Peradeniya, LKA; 4 Medicine, Teaching Hospital Peradeniya, Peradeniya, LKA; 5 Physiotherapy, Kaplan Business School, Sydney Campus, Sydney, AUS; 6 Internal Medicine, Kings Mill Hospital, Sherwood Forest Hospitals NHS Foundation Trust, Sutton-in-Ashfield, GBR; 7 New Initiatives, International Vaccine Institute, Seoul, KOR; 8 Diabetes and Endocrinology, Kings Mill Hospital, Sherwood Forest Hospitals NHS Foundation Trust, Sutton-in-Ashfield, GBR

**Keywords:** glycosylated hemoglobin, randomized clinical trial, type 2 diabetes, snack, salacia reticulata, biscuit

## Abstract

Background

Traditional physicians in Sri Lanka and India use extracts from the plant* Salacia reticulata*,or Kothala Himbutu (KH) to treat diabetes. The effect of a KH biscuit, taken as a snack, on glycemic control was investigated in patients with type 2 diabetes.

Methodology

This triple-blind, randomized, placebo-controlled, two-period, two-sequence, crossover study was conducted in the Internal Medical Clinic, Teaching Hospital Anuradhapura. A research assistant opened computer-generated random numbers enclosed in a sealed envelope and performed treatment allocation. The group outcome was masked from the researchers, patients, and analysts. Both the placebo and the KH biscuit were identical. The primary outcome measure was HbA1c. Intention to treat analyses was used. Glycemic stability was assured in the run-in period, and patients with severe renal, liver, or heart disease were excluded. If patients needed insulin, they were withdrawn from the trial.

Results

From January 2014 to May 2016, 230 patients were screened, and 136 were randomized. Of them, 62 were allocated, 58 completed the placebo biscuit, 71 were allocated, and 69 completed the KH biscuit. After the washout period and crossover, 57 completed the KH and 65 completed the placebo biscuit. The baseline mean HbA1c level was 8.45% (68.9 mmol/mol) and 8.65% (71.0 mmol/mol) for the placebo-KH biscuit and KH-placebo biscuit groups. At the end of the trial, the HbA1c levels in the placebo-KH biscuit group and the KH-placebo biscuit group were 8.23% (66.4 mmol/mol) and 8.53% (69.3 mmol/mol), respectively. The unadjusted mean HbA1c reduced from the baseline with 0.10% (95% CI = -0.12, 0.32) after the placebo biscuit and 0.35% (95% CI = 0.10, 0.60) after the KH biscuit. After the placebo and KH biscuits, the HbA1c values were 8.46% (95% CI = 8.19, 8.73) (69 mmol/mol with 95% CI = 66, 72) and 8.19% (95% CI = 7.90, 8.48) (66 mmol/mol with 95% CI = 63, 69), respectively. The paired sample t-test shows that the reduction was not significant for placebo biscuits (p = 0.324), while it is significant for KH biscuits (p = 0.003). Analysis with multiple imputations confirmed a significant difference between the placebo and KH biscuit in reducing the HbA1c level.

Conclusions

KH biscuit taken as a snack reduces HbA1c by 0.25% compared to placebo without serious renal or liver adverse effects. The biscuit can be safely recommended as a snack to patients with type 2 diabetes.

## Introduction

Recently, there has been increasing interest in herbal medicines. The use of complementary and alternative medicine (CAM) in the United States increased from 34% in 1990 to 42% in 1997. Total visits to CAM practitioners increased by 4,7%, and expenditure on alternative health services increased by 45% between 1990 and 1997 [1]. The National Health & Interview Survey 2012 by the Centers for Disease Control and Prevention found that 17.9% used non-vitamin herbal therapies during the preceding 12 months [2]. In a study on hypoglycemia among type 2 diabetes patients in Sri Lanka, 44 out of 261 patients experiencing hypoglycemia had taken herbs as a food supplement and attributed that to their hypoglycemia [3]. The evidence supporting herbal treatment comes from conventional use rather than data from research trials. Many herbal preparations are used to treat diabetes mellitus in Sri Lanka. There is some evidence from animal studies in humans for the efficacy of herbs used to treat diabetes [4].

Poor glycemic control, multiple medications, adverse reactions, and costs may drive patients toward alternative therapy. *Salacia reticulata* Wight, or “Kothala Himbutu” (KH) in Sinhalese, is a woody climbing plant from the Celastraceae family seen in Sri Lanka and Southeast Asia [5]. Extracts from its root and stem are used to treat diabetes by traditional physicians in Sri Lanka and India [6]. It is also a food supplement for diabetes and obesity in Japan [7]. Traditionally, this preparation is ingested as herbal tea. In a double-blind, randomized, crossover trial, the effectiveness of teabags containing *Salacia reticulata* in reducing blood glucose in persons with type 2 diabetes and its safety was shown in 2006 [8]. The KH biscuit was recently evaluated and proven to have a reduced glycemic index compared to a biscuit without *Salacia reticulata* extract (SRE) [9]. A commercial biscuit containing SRE is already available in the market without adequately assessing its effect on blood sugar. This study aimed to ascertain the effect of the biscuit containing SRE on blood sugar control of type 2 diabetic patients.

## Materials and methods

This was a two-period, two-sequence, crossover trial that was triple-blinded, randomized, and placebo-controlled (AB/BA) with a washout period to minimize the carryover bias.

Protocol changes

The six-point blood sugar series was a secondary outcome measure in the protocol but was later abandoned due to incomplete data. The initial plan was to conduct the trial in two centers, but due to logistical reasons, it was conducted only in one center and sample.

Study participants

Those seeking care for type 2 diabetes at the medical clinic run by the Medicine Department at the Teaching Hospital Anuradhapura were chosen after screening.

Inclusion criteria

Patients with type 2 diabetes diagnosed at least six months before and aged between 30 and 65 years with stable glycemic control (glycosylated hemoglobin (HbA1c) variation of no more than 20%) were included in the study.

Exclusion criteria

Those with an estimated glomerular filtration rate of less than 30 mm/minute/1.73 m^2^ of body surface area, severe symptomatic heart failure or valvular disease, diabetes patients on insulin, and those with liver aspartate transaminase more than 10 times normal were excluded from the study.

Study product

A biscuit (cracker) containing an extract of *Salacia reticulata* Wight (KH biscuit), the proprietary name being “Munchee Kothala Himbutu Biscuit,” was the treatment (http://powo.science.kew.org/taxon/162694-1). This biscuit has been available in the market in Sri Lanka since July 2011 (ingredients detailed in Appendices). For preparation, 210 kg of dry bark from the *Salacia reticulata* Wight plant was boiled repeatedly with water. This solution was evaporated to produce 210 L containing 10.5 kg of dried extract. Then, 100 g of the biscuit mixture and 1.042 mL of the aqueous extract were combined. Thin-layer chromatography fingerprint analysis was used to validate the raw material and was analyzed at the Industrial Technology Institute, Colombo. Both KH and the placebo biscuit were produced by Ceylon Biscuits Ltd. in their factory at Kottawa in the Colombo district and transported to the hospital clinic. Biscuits were packed in identical cardboard boxes and labeled A & B, with four biscuits (22 g; one biscuit weighed 5.5 g) packed in an opaque wrapper. One box had 180 such packets, sufficient for three months. Four biscuits (one pack) were consumed mid-morning, and another four as a mid-afternoon snack.

The KH and placebo cookies had the same calories, carbohydrates, and fiber; 100 g of the KH biscuit contained 447.21 kcal of energy, 10.42 g of protein, 13.97 g of fat (6.29 g saturated), 69.95 g of carbohydrates (less than 0.10 g of sugar), 7.53 g of dietary fiber, and 979 mg of sodium [9]. The KH biscuit had a glycemic index of 30, whereas the placebo biscuit had a glycemic index of 56 [9]. The difference in glycemic index was probably due to SRE in KH biscuits inhibiting enzymes in the digestion of carbohydrates in the human digestive system.

Screening and the run-in period

Consecutive patients who sought or were under treatment for diabetes mellitus at the Teaching Hospital Anuradhapura medical clinic were screened for eligibility, excluding type 1 diabetes patients, and invited. If the fluctuation of HbA1c exceeded 20% during the run-in period (minimum eight weeks), patients were not recruited. The stability of the glycemic control was necessary to prevent excessive variation of HbA1c from interfering with the study outcome, as this was a crossover trial.

Trial process

The trial doctor continued all medications at enrollment and made any required adjustments during the study period (seven months excluding the run-in period). Over the first three months, participants consumed KH or placebo biscuits (depending on the randomization) for mid-morning and afternoon snacks. Then they abstained from eating biscuits for a month during the washout period. The washout period was needed to minimize the carry-over effect. We do not know the half-life of SRE and its impact on glucose metabolism after stopping the biscuit. No formal statistical test was done to analyze the carry-over effect.

Again they consumed biscuits for the following three months till the trial was completed. Patients visited the medical clinic eight to ten times during the study and met the doctor who checked their blood sugar levels and other potential problems at each visit. HbA1c measurements were made at enrollment, baseline (after the run-in period), three months, and study completion. The patient was removed from the research when their blood sugar control worsened, could not be managed with available oral hypoglycemic drugs, and needed insulin.

Biscuits were given to the patients at the start of each study period. Biscuits were not administered under supervision. The patients bought the leftover biscuits when they came for clinic visits. Leftover biscuits were counted to assess compliance.

Outcome variables

The primary outcome variable was glycemic control evaluated with HbA1c carried out centrally [10]. The secondary variable was the adverse renal or liver reactions when consuming the biscuits containing the extract of *Salacia reticulata*.

Sample size 

A method designed specifically for crossover studies was used to determine the sample size at first [11]. The standard deviation (0.9) was taken from baseline data to determine the standard error [8]. The mean difference between the intervention group’s HbA1c and the placebo group was calculated to be 0.5% of HbA1c, which is clinically significant. Due to the small sample size (13 in each arm), we used Pocock’s formula [12] to determine the sample size for parallel investigations. There were 51 patients in each arm when the power (1-beta) was 0.8, and the significance level (alpha) was 0.05. The predicted sample size for a single-center trial was 112 patients, with a 10% expected dropout rate.

Randomization and allocation concealment

An author (SA) used a computer to generate the randomization code (A or B) and inserted them in sealed, serially numbered, opaque envelopes. After the run-in period, the study assistant allocated each eligible participant an appropriate numbered envelope. The study assistant opened the envelope and gave biscuit box A (placebo) or B (KH). The randomization code was in the possession of the producers, Ceylon Biscuits Ltd.

The group outcome was kept a secret from the researchers, patients, and those analyzing the data. The KH biscuit is available in the market. The KH and placebo biscuits were of the same size and had the same packaging, with four biscuits wrapped in opaque paper. The investigators tasted both biscuits and found that KH biscuits had a similar flavor but were slightly darker.

Patients in the study were questioned whether they had ever consumed KH biscuits from the market. This question was to identify potential contamination. Patients were asked about the probable identity of the biscuit (KH biscuit or placebo) after the end of each study period. Their preference for the biscuit type was also recorded at the end of the trial.

Analysis

Two groups were compared at baseline. The difference between the baseline and trial end was calculated for HbA1c values. To overcome the period effect, we used a general linear model (GLM) with repeated measures for the mean difference of HbA1c values after biscuit A and biscuit B. Within-subject (biscuit type), period, and carryover effect, as well as interactions, were investigated. Box’s M tests were used to ensure that the observed covariance matrices of the dependent variables were equal across the two groups. Mauchly’s test was used to verify that the error covariance matrix of the orthonormalized-transformed dependent variables was proportional to an identity matrix. We examined all four types of models, namely, sphericity assumed, Greenhouse-Geisser, Huynh-Feldt, and lower bound, to determine the significance.

We used intention to treat analysis. As the missing data were more than 5%, we examined the dataset to determine whether it showed missing completely at random (MCAR), not missing at random (NMAR), or data missing at random (MAR). A sensitivity analysis was performed to determine whether multiple imputations were required. It included complete case analysis, best-worst-case, and worst-best-case scenario with group mean ± 1SD.

Data and sample collection

Demographic information and recruited patients’ clinical history, investigations, and treatment were entered into a structured questionnaire. HbA1c was determined using ion-exchange high-performance liquid chromatography that was standardized to the National Glycohemoglobin Standardization Program (NGSP) at a lab that was centrally accredited [10]. All other investigations were performed in the same lab.

The data were entered into a structured questionnaire containing demography, patients’ history, treatment, and control of diabetes mellitus. Blood samples were analyzed at a centrally accredited lab, and HbA1c was measured using ion-exchange high-performance liquid chromatography standardized to NGSP [10].

Ethics and clinical trial registration

Ethical clearance was obtained from the Ethics Review Committee of the Faculty of Medicine and Allied Science, Rajarata University of Sri Lanka (ERC/2013/023). Research assistants who were not involved in healthcare delivery to the participants obtained consent. Patients were first provided with a patient information sheet and verbally explained about the study at the screening visit, and written consent was obtained before randomization. This allowed them to discuss their participation with family and significant others. The clinical trial was registered with the National Library of Medicine website (ClinicalTrials.gov) (registration number: NCT02290925).

## Results

Participant flow

**Figure 1:**
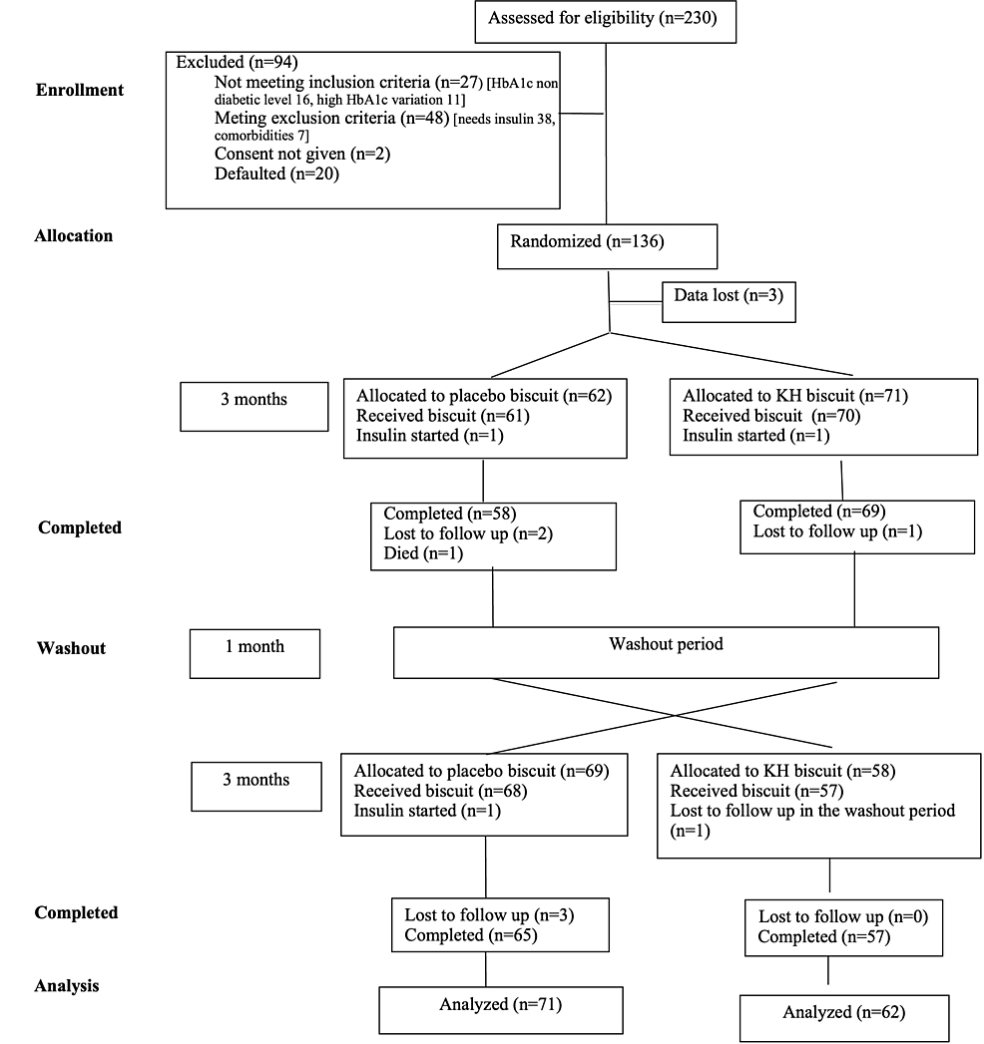
Participant flow. The consort flow diagram of patients with the intention to treat analysis. Data from three patients were missing after randomization. We do not have information about the allocated group for those three patients.

From January 2014 to May 2016, 230 patients with type 2 diabetes were screened. Of them, 94 were excluded, and 136 were randomized (Figure 1).

Of them, only 133 patients received the allocated treatment due to data loss in three patients. We do not know whether these three were allocated to Group 1 or Group 2. The study sample included 40 (30.1%) males and 93 (69.9%) females. The mean age of the participants was 50.5 years (SD = 7.7).

After the baseline assessment, 62 participants were randomly allocated to Group 1 (placebo first and then KH biscuit), and 71 were allocated to Group 2 (KH first and then placebo biscuit). Recruitment was from May 2014 to September 2016. The trial ended in April 2017. At the end of the first period, 58 completed the placebo biscuit (Group 1), and 69 completed the KH biscuit (Group 2). After one month of washout, patients in Group 1 were given KH biscuits, of whom 57 completed, and Group 2 patients were given placebo biscuits, of whom 65 completed. After randomization, there was data loss in three, seven patients went missing (five while on placebo and two while on KH biscuit), three were started on insulin, and one patient died. Death was due to pneumonia during the first period or after one month into the trial while consuming the placebo biscuit. The patient was a 56-year-old healthcare worker with HbA1c of 6.7%, creatinine of 1.01 mg/dL (eGFR of 83 mL/minute), and high urine albumin creatinine ratio (67.6 mg/g) at recruitment.

Protocol violations

Five participants were included in the trial even though their HbA1c value showed a variation of more than 20%. Two persons were excluded based on non-diabetic HbA1c level, but both had HbA1c of 6.5%.

Baseline data

Table 1 shows the baseline clinical and demographic characteristics of the study participants. No significant differences between the two initial groups at baseline were noted. At baseline, no participants were on KH biscuits or other herbal treatments. The mean HbA1c of the sample at the screening was 8.73% (SE = 0.13) (71.9 mmol/mol), and after the run-in period, the baseline value was 8.56% (SE = 0.12) (70.1 mmol/mol).

**Table 1:** Baseline characteristics of the trial participants. KH = Kothala Himbutu

Characteristic	Group 1 (placebo biscuit followed by KH biscuit)	Group 2 (KH biscuit followed by placebo biscuit)
Mean age, years, N (SD)	50.3 (8.4)	50.7 (7.1)
Female, N (%)	42 (67.7)	51 (71.8)
Married, N (%)	57 (91.9)	67 (95.7)
Level of education		
Up to grade 5, N (%)	8 (12.9)	4 (5.7)
Up to grade 10, N (%)	33 (53.2)	49 (70.0)
Completed ordinary level exam or higher, N (%)	21 (32.3)	17 (24.3)
Height, cm, mean (SD)	156 (8.3)	157 (8.3)
Weight, kg, mean (SD)	61.7 (9.0)	60.9 (9.7)
Current diabetes treatment		
Metformin, N (%)	59 (95.2)	63 (88.7)
Sulfonylurea, N (%)	21 (33.9)	42 (59.2)
Sitagliptin, N (%)	1 (1.6)	0
Glycosylated hemoglobin, % (SD)	8.6 (0.2)	8.9 (0.2)
Glycosylated hemoglobin, mmol/mol (SD)	70.5 (2.2)	73.8 (2.2)
Systolic blood pressure, mmHg, mean (SD)	126 (14.0)	127 (16.0)
Diastolic blood pressure, mmHg, mean (SD)	81 (8.1)	82 (8.7)
Albumin creatinine ratio, mg/g, mean (SD)	97.5 (103.9)	90.8 (103.8)
Alanine transaminase, U/L, mean (SD)	38 (20.8)	37 (21.9)
Aspartate transaminase, U/L, mean (SD)	28 (13.9)	28 (15.4)
C-reactive protein, mg/L, mean (SD)	4.6 (5.5)	4.0 (4.5)
Serum creatinine, mg/dL, mean (SD)	0.94 (0.25)	0.94 (0.21)
Estimated glomerular filtration rate, mL/minute, mean (SD)	77.1 (13.4)	76.0 (13.6)

The distribution of HbA1c values at each stage of the trial is shown in Table 2. The baseline mean HbA1c level was 8.45% (68.9 mmol/mol) and 8.65% (71.0 mmol/mol) for the placebo-KH biscuit group and the other group, respectively. At the trial end, the HbA1c level in the placebo-KH biscuit group and the other group was 8.23% (66.4 mmol/mol) and 8.53% (69.3 mmol/mol), respectively. In both groups, a drop after taking the KH biscuit was observed.

**Table 2:** Distribution of HbA1c values in each stage of the trial. KH = Kothala Himbutu

	AB (placebo biscuit first and then KH)				BA (KH biscuit first and then placebo)			
	N	Mean	SE	SD	N	Mean	SE	SD
Screening	62	8.56	0.20	1.54	71	8.87	0.17	1.46
Baseline	62	8.45	0.18	1.40	71	8.65	0.17	1.44
After period 1	57	8.36	0.19	1.43	68	8.16	0.20	1.69
Trial end	57	8.23	0.21	1.58	65	8.53	0.21	1.68

**Figure 2:**
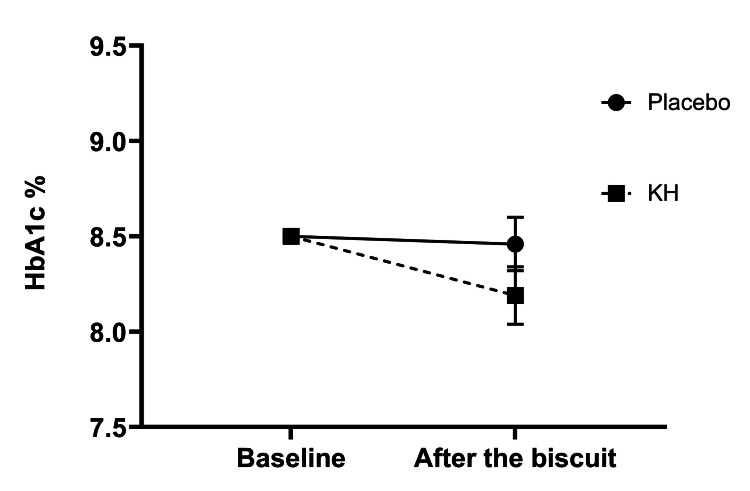
HbA1c values after biscuits A and B. Mean HbA1c values with standard errors after biscuit A (placebo) and biscuit B (Kothala Himbutu (KH) containing an extract of *Salacia reticulata*). The baseline values were fixed at HbA1c 8.5%.

The biscuit effect was analyzed separately to examine the unadjusted effect of the biscuit (Figure 2). The unadjusted mean HbA1c reduced from the baseline with 0.10% (95% CI = -0.12, 0.32) after the placebo biscuit and 0.35% (95% CI = 0.10, 0.60) after the KH biscuit. After the placebo and KH biscuits, the HbA1c values were 8.46% (95% CI = 8.19, 8.73) (69 mmol/mol with 95% CI = 66, 72) and 8.19% (95% CI = 7.90, 8.48) (66 mmol/mol with 95% CI = 63, 69), respectively. The paired sample t-test showed that the reduction was not significant for placebo biscuits (p = 0.324), while it was significant for KH biscuits (p = 0.003).

Of the 136 patients randomized, 122 (89.7%) completed the trial. As the missing data was more than 5%, we examined the data to see whether the missing data could be classified as MCAR. Three patient files were missing after the randomization, even before starting, and one patient each from groups A and B was started on insulin before the intervention. All these could be considered MCAR. However, other patients lost after allocation could not be classified as MCAR. At the same time, there were no indications to conclude that the data were NMAR. Thus, the analysis was based on the assumption of MAR.

Sensitivity analysis

The complete case analysis was done with the last observation carried forward (Table 3). In the GLM repeated measure model, we tested Box’s M tests to ensure that the dependent variable’s observed covariance matrices were equal across the two groups. Box M of 3.39, F 1.11, and a p-value of 0.34 showed that the assumptions were met. Further, we examined Mauchly’s test to verify that the error covariance matrix of the orthonormalized-transformed dependent variables was proportional to an identity matrix. Mauchly’s test value of 1 with a chi-square value of 0 confirmed that the assumptions were met.

**Table 3:** Sensitivity analysis of complete cases versus missing data replaced with the worst-best and best-worst-case scenario. P-values <0.001 are considered significant.

	Source	Sum of squares	df	Mean square	F	P-values
Complete case analysis	Biscuit	7.8	1	7.8	8.57	0.004
	Biscuit type	1.930	1	1.93	2.12	0.148
	Error (biscuit)	119.21	131	0.91		
Worst-best-case analysis	Biscuit	0.06	1	0.06	0.06	0.813
	Biscuit type	1.28	1	1.28	1.24	0.268
	Error (biscuit)	135.91	131	1.04		
Best-worst-case analysis	Biscuit	15.40	1	15.40	14.41	0.000
	Biscuit type	0.47	1	0.47	0.43	0.51
	Error (biscuit)	139.98	131	1.07		

However, the sensitivity analysis showed a significant effect in the best-worst-case scenario, like the complete case analysis. In contrast, the worst-best-case scenario showed a non-significant result. Hence, the complete case analysis was not considered to provide unbiased estimates for this study.

Analysis with multiple imputations

Multiple imputations were done (five imputations) to replace the missing values using a linear regression model using the variables listed in Table 1 and the baseline HbA1c as predictors. Pooled imputation values were used to replace missing data. In the analysis with missing data, Box M of 7.13, F 2.34, and a p-value of 0.071 showed that the assumptions are met. Mauchly’s test value of 1 with a chi-square value of 0 confirmed the previous assumptions. Test of within-subject effect showed that there was a significant difference between biscuit A (placebo) and B (KH biscuit) in reducing HbA1c level (Table 4).

**Table 4:** Effect of biscuit type on HbA1c (Tests of Within-Subjects Effects): analysis with multiple imputations for missing values. P-values <0.001 are considered significant.

Source	Type III sum of squares	df	Mean square	F	P-value
Biscuit type	4.502	1	4.502	6.373	0.013
Biscuit sequence	1.594	1	1.594	2.256	0.136
Error (biscuit)	92.545	131	0.706		

Interactions and period effect

There was no interaction between the biscuit type and the sequence. All four types of models, namely, sphericity assumed, Greenhouse-Geisser, Huynh-Feldt, and lower bound, produced the same results. Test of the period effect resulted in Type III sum of a square and mean square values of 0.027, F 0.007, and p-value of 0.934, showing no period effect on the observed differences.

Participant preference

None of the participants had consumed the KH biscuits before. The responses at the end of each period were combined, and 85 (64%) participants preferred both biscuits, 19 (14%) preferred KH (B), 16 (12%) preferred placebo (A), and the rest (3) neither. Eighty-five (64%) participants believed both types of biscuits contained the active ingredient, 16 (12%) believed KH (B), 14 (11%) believed placebo (A), and the rest (2) neither. Responses from 16 participants were missing.

Adverse effects

Renal and liver functions at the baseline and end of trial periods one and two for each biscuit are shown in Table 5. No deterioration of the renal or liver functions was seen over the study period.

**Table 5:** Renal and liver functions. The glomerular filtration rate was estimated using the Chronic Kidney Disease Epidemiology Collaboration 2009 equation.

Mean (SD) and range of kidney and liver function	Placebo (A) biscuit			Kothala himbutu (B) biscuit		
	Baseline	Period 1	Period 2	Baseline	Period 1	Period 2
Albumin creatinine ratio, mean (SD) (mg/g)	98 (103.9)	62 (69.8)	59 (59.4)	91 (103.8)	66 (66.4)	55 (65.5)
Albumin creatinine ratio, range (mg/g)	6–559	5–337	9–300	8–560	8–300	6–406
Estimated glomerular filtration rate, mean (SD) (mL/minute)	77 (13.4)	75 (13.1)	76 (13.3)	76 (13.6)	75 (13.9)	75 (13.8)
Estimated glomerular filtration rate, range (mL/minute)	41–90	39–90	41–90	41–90	41–90	41–90
Serum creatinine, mean (SD) (mg/dL)	0.94 (0.25)	0.98 (0.22)	0.93 (0.19)	0.94 (0.21)	0.96 (0.21)	0.97 (0.21)
Serum creatinine, range (mg/dL)	0.04–1.84	0.70–1.97	0.61–1.39	0.10–1.47	0.62–1.70	0.60–1.80
Alanine transaminase, mean (SD) (U/L)	38 (20.8)	39 (23.7)	34 (14.7)	37 (21.9)	36 (19.2)	37 (20.3)
Alanine transaminase, range (U/L)	12–95	12–124	12–90	9–129	11–111	11–110
Aspartate transaminase, mean (SD) (U/L)	28 (13.9)	28 (17.9)	24 (9.5)	28 (15.4)	26 (13.1)	27 (14.6)
Aspartate transaminase, range (U/L)	11–97	11–132	10–55	11–111	10–73	12–90

## Discussion

This trial showed that biscuits containing aqueous SRE taken as a snack reduced HbA1c by 0.25% (2.7 mmol/mol) compared to placebo biscuits without serious renal or liver adverse effects. The study was a pragmatic triple-blind randomized clinical trial in a busy general medical clinic. The biscuit can be safely recommended as a snack to diabetes patients. Snacks with carbohydrates are mainly advocated in insulin-treated patients to prevent hypoglycemia [13,14]. However, there are few trials in patients with type 2 diabetes and not on insulin [15].

The KH biscuit with SRE mainly inhibits the α-glucosidase enzymes at the intestinal brush border, breaking down the complex into simple sugars. Available α-glucosidase inhibitors such as acarbose, miglitol, and voglibose are competitive, reversible inhibitors of the intestinal brush border enzymes, and acarbose also inhibits pancreatic amylase. These drugs have modest efficacy but with distressing gastrointestinal (GI) effects [16]. Hence, SRE, one of its active ingredients being a frontline drug for diabetes, is a distant possibility. However, as a snack in patients with diabetes and prediabetes, KH biscuit may be the first choice as the GI effects were minimal.

*Salacia reticulata* and other plants of the *Salacia *genus, such as *Salacia oblonga*, *Salacia prinoides*, and *Salacia chinensis*, have been used in traditional medicine to treat diabetes. Extracts of *Salacia* spp. inhibit carbohydrate digestion in numerous ways to reduce blood sugar. Four enzymes mediate human carbohydrate digestion, namely, salivary, pancreatic α-amylases, maltase glucoamylase (MGAM), and sucrose-isomaltase (SI) enzymes, at the small intestinal brush border. These two enzymes have α-1,4 glucosidase activity and α-1,6 and α-1,2 activity [17]. In 1997, the first member of sulfonium-ion class glucosidase inhibitor salacinol was isolated from an aqueous extract of *Salacia reticulata* [18]. This was followed by four compounds, namely, kotalanol, ponkoranol, salaprinol, and de-o-sulfonated derivatives [17}. With varying potency, these inhibit MGAM and SI in the small intestine. Another compound, mangiferin, in *Salacia reticulata*, impairs fructose 1,6 biphosphatase and glucose 6 phosphatase [19]. Mangiferin also acts as an aldose reductase inhibitor inhibiting glucose flux through the polyol pathway and preventing osmotic damage [20].

Baseline and peak glucose concentrations are unaffected when *Salacia oblonga* extract (SOE) is administered with the main meal. However, the area under the plasma glucose and insulin curve was lower than the test meal. Time is required for the SOE to reach the small intestine to inhibit MGAM, and SI is the likely explanation [21,22]. In this trial, as KH and placebo biscuits were given as mid-morning and mid-afternoon snacks, there was sufficient time to inhibit MGAM and SI before lunch and dinner.

In a PubMed search using the string: “(Salacia [Title/Abstract]) AND diabetes,” 95 articles were extracted, and 15 investigated the effect of *Salacia *species on the glycemic response in humans. Eight were randomized controlled trials (RCTs), but an additional search revealed six more with variable methodological quality [21-29]. Six RCTs were using SOE, three RCTs used *Salacia chinensis* extract, and five included a product containing SRE. One was a sucrose loading test using seven volunteers, and SRE reduced glucose spikes [30]. Twenty patients with fasting hyperglycemia were randomized and given SRE for six weeks in a crossover study with an average decrease of HbA1c of 0.11% and a decrease in body mass index [31]. A significant decrease of 0.54% in HbA1c was seen in another crossover RCT in 51 patients with type 2 diabetes using tea bags with SRE for three months [8]. There was a significant reduction of blood glucose and lipids in 29 patients in a double-blind crossover RCT after giving SRE for six weeks [32]. In a randomized open-label trial, SRE with vitamin D for six weeks reduced weight [33].

Only three of the 13 RCTs used HbA1c [8,24,31]. Except for one study (1-14 days), none of the RCTs had a washout period between the placebo and the study product [21]. The largest RCT recruited 82 patients [23]. The maximum duration was three months [8]. There were three RCTs where *Salacia* extracts were successfully tested as a food supplement [21,22,31], two RCTs as tea [8,24], and two RCTs as a commercial food product [24,33]. In this study, centralized HbA1c measurement was performed with a run-in period before the randomization to ensure stable HbA1c. There was a washout period, and 133 patients were followed up for three months.

Several animal studies have evaluated the safety of the extracts from *Salacia* species. Adverse pregnancy outcome was observed in Wistar rats but was found to be mediated through low glycemic levels causing intrauterine growth retardation [34]. A genotoxicity study showed weakly positive human chromosomal aberrations with SOE but without a dose relationship [35]. However, a study using more than 10 times the human dose found no adverse effect [36]. Several animal studies have found that *Salacia* extract improves non-alcoholic fatty liver disease without hepatotoxicity [37]. Renal and liver parameters were measured throughout and no adverse effects were noted.

Although the KH biscuit’s slightly altered flavor and deeper color may have interfered with the patient’s ability to remain blind, participants’ beliefs about the biscuit containing active ingredients revealed that they could not identify the KH biscuit.

The KH biscuit as a treatment for type 2 diabetes needs further research. However, we can expect only a modest effect on HbA1c because the unadjusted decrease due to the active biscuit was slight (0.35%). A more systemic inquiry into the GI side effects is needed if a larger dose of SRE in a tablet or a capsule is to be tried in a future randomized controlled trial.

Limitations

Losing all data on three patients, including the allocated group, was a drawback. Removing patients on insulin and with a variation of HbA1c of more than 20% affected the pragmatic nature of the study. The effect of KH biscuit on serum lipids and weight was not checked systematically. Change in insulin sensitivity was not assessed. We did not check the GI side effects of biscuit consumption systematically. Biscuit consumption was verified by counting the biscuits and interviewing the patients. The formal tests of undigested carbohydrates, such as hydrogen breath tests, were not employed.

## Conclusions

This study was a placebo-controlled, two-period, two-sequence, crossover study (AB/BA) with a washout period. This was also a pragmatic, triple-blind, randomized clinical trial in a busy general medical clinic, with 90% of participants randomized completing the trial. No period effect, interaction, or missing data affected the study outcome. KH biscuit as a snack improves glycemic control compared to a placebo without serious renal or liver adverse effects. The biscuit containing SRE extract inhibits the α-glucosidase enzymes at the intestinal brush border, breaking the complex carbohydrates into simple sugars without distressing GI effects. These biscuits can be safely recommended as a snack to patients with type 2 diabetes. The KH biscuit as a treatment for type 2 diabetes needs further research. However, we can expect only a modest effect on HbA1c because the unadjusted decrease due to the active biscuit was slight (0.35%).

## Appendices

**Table 6:** Contents of the study biscuit. The placebo biscuit was the same except for the water extract of Kothala Himbutu.

Name of ingredient	Quantity (%)
Wheat flour	73.51
Yeast	1.06
Vegetable fat	12.82
Wheat bran	8.12
Leavening agents	0.82
Salt	1.48
Soya lecithin	0.15
Water extract of Kothala Himbutu	
